# Changes in blood pressure according to stature in Mexican adults

**DOI:** 10.11606/s1518-8787.20210550032531

**Published:** 2021-11-18

**Authors:** Miguel A. Perez-Sastre, Luis Ortiz-Hernandez

**Affiliations:** I Universidad Nacional Autónoma de México Ciudad de México México Universidad Nacional Autónoma de México. Programa de Maestría y Doctorado en Ciencias Médicas y Odontológicas y de la Salud. Ciudad de México, México; II Universidad Autónoma Metropolitana unidad Xochimilco Departamento de Atención a la Salud Ciudad de México México Universidad Autónoma Metropolitana unidad Xochimilco. Departamento de Atención a la Salud. Ciudad de México, México

**Keywords:** Adult, Blood Pressure, Anthropometry, Hypertension, Risk Factors, Longitudinal studies

## Abstract

**OBJECTIVE:**

To determine the possible existence of differences in blood pressure change over time according to stature in Mexican adults.

**METHODS:**

We analyzed the National Household Living Standards Survey databases following household members between 2005 and 2009. We selected participants who were between 20 and 40 years old (n = 7,130). We estimated multilevel models with random intercept to analyze differences in blood pressure changes according to stature. We adjusted the models for age, locality size, geographic region, *per capita* family income, waist-to-height ratio, physical activity, alcohol consumption, smoking, and use of antihypertensive drugs.

**RESULTS:**

In both sexes, baseline blood pressure tended to be lower as stature decreased. The differences were maintained in both the crude and adjusted models. In men, the increases in systolic pressure over time tended to be higher as stature increased.

**CONCLUSIONS:**

Contrary to what studies observed in high-income countries, in Mexico blood pressure was positively associated with stature.

## INTRODUCTION

Arterial hypertension is highly prevalent in Mexico, with almost one third of adults suffering from it in 2012^1^. It is the most common condition in primary care; in 2010, it was the main cause of death and disability adjusted life years. It is a risk factor for several of the main causes of mortality^2^. This contrasts with the fact that it is a preventable risk factor.

In recent years, cohort studies conducted in mainly European and Brazilian populations have established a negative association between stature and the risk of developing cardiovascular disease^3–7^. In different populations, an inverse association has been established between blood pressure and stature, tending to be higher in those who are shorter^4,5,8–11^.

These studies are consistent with the hypothesis proposed by Barker, who postulated that adverse conditions during prenatal life and childhood increase the risk of suffering chronic diseases in adulthood^12^. This theory holds that adversities present during early stages of life produce structural and physiological modifications in the organism. Part of these changes result in short stature; those who have deficiencies during development generally reach shorter stature in adulthood^3,5^.

It possible to explain the inverse association between stature and blood pressure by different mechanisms: in shorter individuals the diameter of the blood vessels is smaller^13,14^; they have a smaller number of capillaries and less elasticity of the large vessels^14^; as well as greater arterial intima-media thickness and greater incidence of atherosclerosis^14^. These mechanisms contribute to a decrease in vascular lumen, increasing peripheral resistance^13,14^.

The physical characteristics of the Mexican population are not comparable with the studied populations; therefore, it is not possible to assume that the results of these studies are valid for this population. For example, the average stature in Mexico is 156.8 and 169.0 cm for women and men, respectively; while in Brazil it is 160.9 and 173.6 cm; and in the United Kingdom it is 169.5 and 177.5 cm^15^. This characteristic is similar with populations from other Latin American countries such as Colombia, Honduras and Bolivia, which have an average stature similar to that of Mexico^16^. Differences in stature are probably the result of genetic characteristics and living conditions. Genetically, the Mexican population differs from the Brazilian and European populations because, although our population is a mixture of Amerindian, European and African genes, the Amerindian component predominates^17^. In addition to the above, there are genetic differences (higher proportion of indigenous population), differences in living conditions (less human development) and feeding patterns (lower consumption of foods of animal origin) making the Mexican population different from that studied populations^16^.

On the other hand, in Mexico, around 1.5 million children under five years old were chronically undernourished in 2012^1^, favoring short stature in adulthood^5^. Therefore, this study sought to determine the existence of differences in changes in blood pressure over time according to stature in Mexican adults, considering the hypothesis that individuals of shorter stature will have a greater increase in blood pressure.

## METHODS

To achieve the proposed objective, we carried out a secondary analysis of the databases of the National Household Living Standards Survey (ENNViH - *Encuesta Nacional sobre Niveles de Vida de los Hogares*) conducted in Mexico. ENNViH is a cohort study following a sample of households with three surveys: 2002 (ENNViH-1), between 2005 and 2006 (ENNViH-2), and between 2009 and 2012 (ENNViH-3). During the follow-ups (2005 and 2009), the participants of the initial round were contacted again. The sample of the ENNViH has national, rural-urban and regional representativeness. This study used data from the second and third surveys; the 2005 information corresponds to our baseline measurement and the 2009 information corresponds to the follow-up. The ENNViH questionnaires were adapted to the Mexican population, and the ethics committee of the National Institute of Public Health approved the study. The informed consent was obtained from all participants^18^.

For the analysis, we included individuals between 20 and 40 years old at the beginning of the study (ENNViH-2, n = 8,106), considering that during this stage of life, stature remains stable. Those who, in baseline measurement (2005), were pregnant or breastfeeding (n = 323); those who did not have systolic or diastolic blood pressure record (n = 312) or whose values were outside the biologically possible range (n = 17); those who did not have a record of stature (n = 19); those whose sex was not specified (n = 201); and those whose follow-up time did not correspond to the time period in which the data were collected (n = 104) were excluded. Follow-up time was obtained from the age difference between the second and first cycle. The shortest follow-up was two years for those surveyed during 2006 after the day and month of their birth, with follow-up during 2009 before the day and month of their birth; while the longer was eight years for those surveyed during 2005 before the day and month of their birth, with follow-up during 2012 after the day and month of their birth. An analytical sample of 7,130 individuals was formed.

During follow-up, 81.1% of the participants included in the initial round were contacted. However, blood pressure measurement was only recorded for 62.7% (n = 4,472). Women who were pregnant or breastfeeding during the second time (n = 202) were not excluded, but only their baseline information was included for analysis, considering them as lost during follow-up. The comparison between those who had complete follow-up information with those who only had information from the first round showed that those who were followed up in general had short stature and higher blood pressure (Supplementary Table 1). Due to the type of analysis, we did not exclude losses in follow-up and those who had missing information, since the results of their first measurement were available.

We measured the blood pressure in the manner established in the guidelines for the detection of arterial hypertension, with the participants seated in a quiet environment after at least five minutes of rest, with the arm at heart level, recording two measurements in each participant^2^, obtaining the results by means of an OMRON model HEM^19^ digital baumanometer, recorded in mmHg. We used the average of the two measurements for the analysis. We considered as implausible values those with measurements ≤ 60 or ≥ 300 mmHg for systolic and ≤ 30 or ≥ 150 mmHg for diastolic. We calculated the change in blood pressure by subtracting the values of the first moment from those of the second moment.

Specialized personnel trained for this activity, following standardized procedures^20^, obtained the anthropometric measurements. We measured the stature using a SECA wall stadiometer with a precision of 1.0 mm, with the individual standing barefoot, resting heels, calves, buttocks, back and head on the wall, reporting the results in centimeters and calculating quintiles for each sex.

The covariates considered were the participants’ age, socioeconomic position, as well as habits related to health and an indicator of adiposity, the geographic region and the size of the town where they lived. Age was taken in completed years recorded in the questionnaire and was grouped into five categories: 20.0–24.9 years old, 25.0–29.9 years old, 30.0–34.9 years old, 35.0–39.9 years old and 40.0 years old or more. To measure the socioeconomic position of the participants, we considered family income and parents’ schooling. We obtained the *per capita* family income from the sum of the income of the family members (business, rent, labor, rural and transfers from another family member or person). We divided the total by the number of household members and reported in Mexican pesos. On the other hand, when data was available, we analyzed the father’s schooling; and when it was not available, we considered the mother’s schooling.

For habits (physical activity, alcohol consumption, smoking and use of antihypertensive drugs), dichotomous variables were generated, distributing the sample as positive or negative according to the responses. We considered physical activity positive for who reported that performs physical activity regularly. In addition, we classified as positive for alcohol consumption those who mentioned they usually drink alcoholic beverages in any of the situations mentioned in the questionnaire. In smoking, likewise, those who reported smoking or having smoked in the past were positive. Those who mentioned being hypertensive and under treatment were classified as positive for the use of antihypertensive drugs. We measured waist circumference with a fiberglass measuring tape 200 cm long and accurate of one decimeter, with the individual standing upright, feet together, abdomen relaxed and arms crossed in front of the chest. The anatomic reference was the iliac crest and the last rib. The measurement was reported in centimeters^20^. We calculated the waist-height index (WHI) by dividing waist circumference by stature. We considered it as an indicator of adiposity, since it uses to be a good predictor in the evaluation of cardiovascular risk. It was considered as a possible mediator of the relationship between stature and blood pressure, since people of shorter stature tend to have greater adiposity and, in turn, adiposity is one of the strongest predictors of blood pressure^21^.

We divided the regions according to the national development plan 2001–2006^22^, resulting in four regions: center, midwest, south-southeast and north (including northeast and northwest). We formed four categories according to the size of the locality: > 100,000; 15,001–100,000; 2,500–15,000; and < 2,500 inhabitants.

We used the Stata version 14 program for database cleaning and statistical analysis. We considered the expansion factor at the individual level to obtain weighted relative frequencies of the categorical variables for each moment in each sex. We used ANOVA testing to look for differences according to stature in baseline blood pressure and changes over time.

We used multilevel models^23^, considering participant identification as a random intercept, to look for differences in blood pressure (baseline and over time) according to stature. With multilevel models it is recognized that there is intra-subject correlation. The interaction of the cycle with stature allows us to recognize between cross-sectional and longitudinal differences. The intraclass correlation coefficient for systolic and diastolic pressure in men was 0.20 and 0.16; while in women it was 0.25 and 0.18, respectively. The cycle, which identifies to which round each measurement belongs, was included as a time variable. We added the quadratic term of stature and its interaction with cycle in each of the models to evaluate the quadratic effect of stature. We used crude models adjusted for sociodemographic data, WHI and health-related habits (physical activity, alcohol consumption, smoking and use of antihypertensive drugs). The parental schooling variable had a high proportion of missing data (women, 15.8%; men, 18.2%), so models were estimated with and without it.To improve the understanding of the results, marginal means were estimated and plotted from the fitted models^a^. We performed the analysis stratifying by sex.

## RESULTS

Women participants were 56.1%. The mean age was 29.5 years at first, increasing to 35.0 years during follow-up. The average follow-up time was 4.3 years.

In both sexes, half of the participants were between 30 and 39 years old during 2005 (Table 1). For the second time, the majority were over 35 years old. Most of the participants lived in cities and were located mainly in the central region. In both cycles, a higher proportion of the population did not practice physical activity or smoke. The proportion of men who were physically active, smoked or consumed alcohol was higher than the proportion of women. At follow-up, in men, the proportion of smokers increased, while alcohol consumption decreased. In women both, the proportion of smokers and alcohol consumption increased.

**Table 1:** Characteristics of the Mexican population between 20 and 45 years old^a^ in 2005 and 2009.

		2005		2009	
		Men (n = 3,133)	Women (n = 3,997)	Men (n = 1,734)	Women (n = 2,536)
		%	%	%	%
Age					
	20 to 24 years old	26.9	23.4	3.8	2.4
	25 to 29 years old	19.5	19.9	22.2	19.0
	30 to 34 years old	26.3	26.7	20.1	18.6
	35 to 39 years old	23.0	24.3	25.3	30.6
	40 years old or older	4.3	5.8	28.5	29.3
Stratum					
	> 100,000	49.3	47.4	48.5	47.7
	15,001–100,000	12.4	12.6	14.8	14.0
	2,500–15,000	14.7	14.1	14.2	13.5
	< 2,500	23.6	25.9	22.5	24.8
Geographic Region					
	Center	32.1	33.4	30.6	30.5
	South-Southeast	21.7	22.4	23.0	23.6
	Midwest	23.7	21.9	25.5	23.2
	North	22.5	22.3	20.9	22.7
Physical activity					
	Positive	20.0	11.5	22.9	17.2
Smoking					
	Positive	23.2	8.0	26.9	9.1
Alcohol consumption					
	Positive	61.4	18.8	58.5	24.7
		x¯ (SD)	x¯ (SD)	x¯ (SD)	x¯ (SD)
Household income*per capita*		14,268 (23,103)	13,546 (50,358)	15,105 (17,034)	13,332 (20,340)
P. systolic		116.1 (11.7)	111.2 (11.9)	123.7 (12.9)	112.9 (15.3)
P. diastolic		76.2 (9.7)	72.9 (9.7)	80.1 (9.4)	76.9 (10.9)
Stature		167.2 (7.6)	154.7 (6.9)	166.6 (7.0)	153.9 (6.8)

n: sample participants; %: weighted estimate; x¯: weighted average; SD: weighted standard deviation; P: pressure

a The age limit of 45 years covers the age of the participants at the time of follow-up.

*Per capita*family income was higher in men than in women in both cycles, but while in men an increase in average income is present, in women it decreased slightly. The average systolic and diastolic pressure in both years was higher in men than in women. Similarly, the increase in systolic pressure among men was greater than in women, while the increase in diastolic pressure was slightly greater in women. In men, the average stature was greater than in women, by 12.5 cm. In 2009 the average stature in women decreased by 0.8 cm, while in men it decreased by 0.6 cm.

In both sexes, baseline blood pressure was lowest in the shorter stature quintile, while it was highest in the taller stature quintile (Table 2). However, the differences were significant only in men, where blood pressure tended to be lower as the stature quintile was shorter.

**Table 2:** Baseline values and changes between 2005 and 2009 in systolic and diastolic blood pressure according to stature in ENNViH participants aged 20 to 45 years old^a^.

		Man				Women			
		Systolic		Diastolic		Systolic		Diastolic	
		Baseline	Δ	Baseline	Δ	Baseline	Δ	Baseline	Δ
		M	M	M	M	M	M	M	M
Stature									
	QV	**118.4** b	8.4	**77.5** b	3.4	112.2	1.0	73.5	3.4
	QIV	**118.7**	6.2	**76.8**	4.1	112.3	1.2	72.7	4.6
	QIII	**116.2**	7.6	**76.4**	3.4	111.1	2.1	72.9	4.0
	QII	**116.8**	8.6	**75.8**	4.9	111.5	2.2	72.3	4.8
	QI	**115.5**	7.3	**75.2**	4.0	110.2	2.3	71.9	4.5

M, mean; Baseline, value in 2005; Δ, change between 2005 and 2009; bold values imply significant differences in pressure between at least two stature quintiles.

a The age limit of 45 years covers the age of the participants at the time of follow-up.

b p < 0.001.

In each of the multilevel models (Tables 3 and 4), in both sexes, systolic and diastolic pressures were higher at follow-up compared to baseline measurement, except for systolic pressure in women, where this difference was significant only in the crude model (model 0). In men (Table 3 and supplementary figure), both pressures, at the start of follow-up, tended to be lower as stature became shorter. We observed this trend both in the crude models and in those adjusted for different covariates (models 0 to 3). When adjusted for WHI this relationship became stronger (model 2), both in systolic and diastolic. In evaluating the quadratic effect, we identified the increases in systolic pressure over time tending to be higher as stature increased, in each of the models (models 0 to 3). The effect on diastolic pressure was similar but only when adjusting for WHI and all variables (models 2 and 3).

**Table 3:** Multilevel models considering systolic and diastolic blood pressure as dependent variables and stature as independent variable in men aged 20 to 45 years^a^ participating in the ENNViH (2005, 2009).

		Mod. 0		Mod. 1		Mod. 2		Mod. 3	
		β	S.E	β	S.E	β	S.E	β	S.E
Systolic									
	Stature	**0.143**	**0.030**	**0.111**	**0.031**	**0.151**	**0.030**	**0.159**	**0.032**
	Stature*stature	-0.003	0.003	-0.002	0.003	-0.002	0.003	-0.001	0.003
	Cycle	**7.487**	**0.425**	**6.460**	**0.452**	**5.940**	**0.449**	**5.978**	**0.467**
	Cycle*stature	0.073	0.048	0.064	0.048	0.065	0.047	0.028	0.049
	Cycle*stature*stature	**0.009**	**0.004**	**0.009**	**0.004**	**0.009**	**0.004**	**0.010**	**0.004**
Diastolic									
	Stature	**0.099**	**0.023**	**0.090**	**0.024**	**0.129**	**0.024**	**0.122**	**0.025**
	Stature*stature	-0.002	0.002	-0.002	0.002	-0.002	0.002	-0.002	0.002
	Cycle	**3.785**	**0.342**	**2.914**	**0.361**	**2.375**	**0.356**	**2.320**	**0.370**
	Cycle*stature	-0.016	0.039	-0.018	0.039	-0.018	0.038	-0.023	0.040
	Cycle*stature*stature	0.006	0.004	0.006	0.004	**0.006**	**0.004**	**0.008**	**0.004**

β, Regression coefficient; S.E., standard error. Bold values indicate significant differences. For main effects we used a significance level of 0.05 (p < 0.05) and for interactions the significance level was 0.10 (p < 0.10). Mod. 0, model unadjusted for other variables. Mod. 1, model adjusted for age, stratum, region and per capita family income. Mod. 2, model adjusted for the variables of model 1 and waist-height index. Mod. 3, model adjusted for the variables of model 2 plus physical activity, alcoholism, smoking, and use of antihypertensive drugs.

a The limit of 45 years covers the age of the participants at the time of follow-up.

In women (Table 4 and supplementary figure), baseline pressures were also lower as stature was shorter, especially after adjusting for WHI and habits related to health (models 2 and 3). No differences in changes over time were identified, nor when using quadratic terms (Supplementary table 2).

**Table 4:** Multilevel models considering systolic and diastolic blood pressure as dependent variables and stature as independent variable in women aged 20 to 45 years^a^ participating in the ENNViH (2005, 2009).

		Mod. 0		Mod. 1		Mod. 2		Mod. 3	
		β	S.E	β	S.E	β	S.E	β	S.E
Systolic									
	Stature	**0,069**	**0,027**	**0,085**	**0,029**	**0,163**	**0,029**	**0,165**	**0,030**
	Cycle	**2,394**	**0,328**	0,428	0,348	0,156	0,345	0,298	0,355
	Cycle*stature	-0,062	0,049	-0,062	0,049	-0,045	0,048	-0,039	0,049
Diastolic									
	Stature	0,034	0,021	**0,045**	**0,022**	**0,116**	**0,023**	**0,113**	**0,023**
	Cycle	**4,677**	**0,240**	**3,510**	**0,259**	**3,219**	**0,257**	**3,292**	**0,263**
	Cycle*stature	-0,048	0,035	-0,045	0,035	-0,028	0,034	-0,029	0,035

β, Regression coefficient; S.E., standard error. Bold values indicate significant differences. For main effects we used a significance level of 0.05 (p < 0.05) and for interactions the significance level was 0.10 (p < 0.10). Mod. 0, model unadjusted for other variables. Mod. 1, model adjusted for age, stratum, region and *per capita* family income. Mod. 2, model adjusted for the variables in model 1 and waist-height index. Mod. 3, model adjusted for the variables of model 2 plus physical activity, alcoholism, smoking and use of antihypertensive drugs.

a The 45-year limit covers the age of the participants at the time of follow-up.

The results of the models when adjusting for parental schooling (Supplementary tables 3 and 4) did not differ from those obtained without including it (Tables 3 and 4). Therefore, the manuscript reports only the latter.

## DISCUSSION

Considering the epidemiological evidence from other populations^3–5,8–11^ and the anatomical and physiological changes described^13,14^, an inverse relationship between stature and baseline blood pressure would be expected. However, in Mexican adults, baseline blood pressure tended to be higher as stature was taller in both sexes, with wider differences among men than among women. In the changes in blood pressure, we found differences only in men when evaluating the quadratic effects; contrary to expectations, as stature increased, the increase in blood pressure was higher.

Differences in the genetic composition of the population could help explain why the behavior of blood pressure was not as expected. The ethnic mix of our country resulted from the combination of three ancestries: Amerindian, European and African. European ancestry has been associated with taller stature^17^ as well as with a higher risk of presenting cardiovascular disease^24^ compared to Amerindian ancestry. On the other hand, Amerindian ancestry is associated with a greater probability of having short stature^25^. In other words, it is possible that the Euro-descendant population in Mexico is the one that is taller and has higher blood pressure than the rest of the population, and this is what explains the positive relationship between these two variables observed in our analysis. Unfortunately, the ENNViH did not measured markers of ancestry, so it is not possible to evaluate this possibility. On the other hand, the relationship between blood pressure and stature may not be linear, so that in populations of smaller stature compared to European countries, it may be contrary to that reported in populations of taller stature.

In addition to genetics, diet quality could be another element that helps to explain why taller stature participants had higher blood pressure. In Mexico, the availability and consumption of ultra-processed foods differ according to geographic region and socioeconomic level (similar to what occurs with stature), with higher consumption in the north and by people with a higher socioeconomic level^26^. Ultra-processed foods have a higher sodium content compared to those of natural origin^26,27^, which explains why their consumption is associated with elevated blood pressure^27^. Therefore, it is possible that people with taller stature tend to have a higher socioeconomic level^28^ and thus may have a higher consumption of ultra-processed foods, favoring higher blood pressure as stature increases. We did not explore this possibility, since ENNViH has no information related to diet.

We confirmed the participation of WHC as a mediator in the relationship between stature and blood pressure. WHC was inversely related to stature; those with shorter stature had higher WHC, as reported in a study conducted in southern Mexico^25^. In the models where it was not included, the differences according to stature were smaller but, adding to the models, the relationship of stature with baseline systolic and diastolic pressure was stronger^21^.

Among the limitations of the study, it is important to point out that ENNViH was not designed to test our hypothesis, so it was necessary to adapt the available information. In addition, with the exception of anthropometric measurements, we obtained the information using questionnaires. The average follow-up time of this study was four years, while several of the studies conducted have a follow-up time of more than 15 years^5,6,11^. Finally, blood pressure changes over time may be significant at another stage of life. That is, differences in blood pressure changes were probably not identified because the follow-up period was at a time when blood pressure rises uniformly, regardless of stature. It is possible to identify the differences at earlier stages^6^ or at later ages than those included in the investigation^41^.

Despite the aforementioned limitations, this study provided an overview of the association between stature and blood pressure in the Mexican population. We measured the blood pressure according to the recommendations of the guidelines for the detection of hypertension^2^. Although it may be useful to have more than two measurements^29^, the increase in the number of measurements has been of minimal benefit in the detection of cardiovascular risk^30^. In addition, we analyzed data from a nationally representative study with a longitudinal design. The use of multilevel models made it possible to distinguish between cross-sectional differences in changes over time. Therefore, the relationships found are causally stronger than those that could have been found in a cross-sectional study.

In summary, in the Mexican population, the effect of stature on blood pressure was contrary to the expected, since it was positively related. In the context of the epidemiological transition, the predominance of chronic undernutrition in Latin American populations could be a factor that increases the burden associated with chronic diseases. Our analysis shows that this may not be entirely true. In contrast to the above, in Mexican adults, blood pressure is related in the expected direction to traditional risk factors such as adiposity. Therefore, in order to address the problem of arterial hypertension and its consequences in the country, it is necessary to continue investigating other environmental, genetic and dietary factors, as well as to address the modifiable factors already known. It would be advisable to carry out studies with a similar design, but at different stages of life, to evaluate the blood pressure and identify the times when it shows a greater change.

## Footnotes

a Supplementary figure may be requested to the authors.
