# Supplementary material for: Changes in blood pressure according to stature in Mexican adults
Source: Rev Saude Publica. 2021 Nov 18;55:87. doi: 10.11606/s1518-8787.20210550032531 (PMC8647983; doi:10.11606/s1518-8787.20210550032531)
Supplement: Translation

# Translation: Cambios en la presión arterial de acuerdo con la estatura en adultos mexicanos

**Authors:** Miguel A. Perez-Sastre, Luis Ortiz-Hernandez

**Affiliations:** I Ciudad de México México Universidad Nacional Autónoma de México. Programa de Maestría y Doctorado en Ciencias Médicas y Odontológicas y de la Salud. Ciudad de México, México; II Ciudad de México México Universidad Autónoma Metropolitana unidad Xochimilco. Departamento de Atención a la Salud. Ciudad de México, México

**Keywords:** Adulto, Presión Arterial, Antropometría, Hipertensión, Factores de Riesgo, Estudios Longitudinales

## Abstract

**OBJETIVO:**

Determinar si existen diferencias en el cambio de la presión arterial a lo largo del tiempo de acuerdo con la estatura en adultos mexicanos.

**MÉTODOS:**

Se analizaron las bases de datos de la Encuesta Nacional de Niveles de Vida de los Hogares en la que se siguieron a los miembros de los hogares entre el 2005 y 2009. Se seleccionaron a los participantes que tenían entre 20 y 40 años (n = 7,130). Se estimaron modelos multinivel con intercepto aleatorio para analizar diferencias en los cambios de la presión arterial de acuerdo con la estatura. Los modelos fueron ajustados por edad, tamaño de localidad, región geográfica, ingreso familiar *per cápita*, índice cintura-estatura, actividad física, consumo de alcohol, tabaquismo y uso de antihipertensivos.

**RESULTADOS:**

En ambos sexos, la presión arterial inicial tendió a ser menor conforme la estatura fuese menor. Las diferencias se mantuvieron tanto en los modelos crudos como en los modelos ajustados. En los varones los incrementos de la presión sistólica a lo largo del tiempo tendieron a ser mayores conforme la estatura era más alta.

**CONCLUSIONES:**

Contrario a lo observado en países de altos ingresos, en México la presión arterial se asoció positivamente con la estatura.

## INTRODUCCIÓN

La hipertensión arterial en México tiene alta prevalencia, casi un tercio de los adultos la padecía en el 2012^1^. Es la condición más común en la atención primaria, en el 2010 fue la principal causa de muerte y de discapacidad ajustada por años de vida. Es un factor de riesgo para varias de las principales causas de mortalidad^2^. Lo anterior contrasta con el hecho de que es un factor de riesgo prevenible.

En los últimos años, mediante estudios de cohorte desarrollados en poblaciones principalmente europeas y de Brasil, se ha establecido una asociación negativa entre la estatura y el riesgo de desarrollar enfermedades cardiovasculares^3–7^. En diferentes poblaciones se ha establecido una asociación inversa entre la presión arterial y la estatura, tendiendo a ser mayor en quienes tienen menor estatura^4,5,8–11^.

Estos estudios hacen eco a la hipótesis planteada por Barker, quien postuló que las condiciones adversas durante la vida prenatal y la infancia aumentan el riesgo de padecer enfermedades crónicas en la edad adulta^12^. Esta teoría sostiene que las adversidades presentes durante etapas tempranas de la vida producen modificaciones estructurales y fisiológicas en el organismo. Parte de estos cambios se traducen en estatura baja, quienes tienen deficiencias durante el desarrollo generalmente alcanzan menor estatura en la edad adulta^3,5^.

La asociación inversa entre estatura y presión arterial podría explicarse por diferentes mecanismos: en individuos de menor estatura el diámetro de los vasos sanguíneos es más pequeño^13,14^; tienen menor número de capilares y menor elasticidad de los grandes vasos^14^; así como mayor grosor de la íntima media arterial y mayor afectación por aterosclerosis^14^. Estos mecanismos contribuyen a la disminución del lumen vascular, incrementando las resistencias periféricas^13,14^.

Las características físicas de la población mexicana no son comparables con las poblaciones que han sido estudiadas, por lo tanto, no es posible asumir que lo observado se puede extrapolar a esta población. Por ejemplo, la estatura promedio en México es de 156.8 y 169.0 cm para mujeres y hombres, respectivamente; mientras que en Brasil es de 160.9 y 173.6 cm; y en Reino Unido es de 169.5 y 177.5 cm^15^. Esta característica es compartida con poblaciones de otros países latinoamericanos como Colombia, Honduras y Bolivia, las cuales tienen estatura promedio similar a la de México^16^. Las diferencias en estatura, probablemente, son producto de factores genéticos y condiciones de vida. Genéticamente, la población mexicana difiere de la población brasileña y poblaciones europeas debido a que nuestra población, aunque es una mezcla entre genes amerindios, europeos y africanos, predomina el componente amerindio^17^. Sumado a lo anterior, existen diferencias genéticas (mayor proporción de población indígena), en condiciones de vida (menor desarrollo humano) y patrones de alimentación (menor consumo de alimentos de origen animal) que hacen que la población mexicana sea distinta a las poblaciones que han sido estudiadas^16^.

Por otra parte, en México, alrededor de 1.5 millones de niños menores de cinco años presentaban desnutrición crónica en el 2012^1^, lo que favorece tener estatura baja en la edad adulta^5^. Por lo tanto, este estudio buscó determinar si existen diferencias en los cambios de la presión arterial a lo largo del tiempo de acuerdo con la estatura en adultos mexicanos, teniendo como hipótesis que los individuos de menor estatura tendrán mayor incremento en la presión arterial.

## MÉTODOS

Para alcanzar el objetivo planteado, se realizó un análisis secundario de las bases de datos de la Encuesta Nacional sobre Niveles de Vida de los Hogares (ENNViH) realizada en México. La ENNViH es un estudio de cohorte que dio seguimiento a una muestra de hogares con tres levantamientos: 2002 (ENNViH-1), entre 2005 y 2006 (ENNViH-2), y entre 2009 y 2012 (ENNViH-3). Durante los seguimientos (2005 y 2009) se contactó de nuevo a los participantes de la ronda inicial. La muestra de la ENNVIH tiene representatividad nacional, rural-urbana y regional. Para este estudio, se utilizaron los datos del segundo y tercer levantamiento, la información del 2005 corresponde a nuestra medición basal y la del 2009 al seguimiento. Los cuestionarios de la ENNViH fueron adaptados a la población mexicana, el estudio fue aprobado por el comité de ética del Instituto Nacional de Salud Pública. Se obtuvo consentimiento informado de cada uno de los participantes^18^.

Para el análisis se incluyeron a los individuos que tenían entre 20 a 40 años durante el primer momento (ENNViH-2, n = 8,106), considerando que durante esta etapa de la vida la estatura permanece estable. Se excluyeron a quienes en la medición basal (2005) se encontraban embarazadas o en periodo de lactancia (n = 323); no contaban con registro de presión sistólica o diastólica (n = 312) o tenían valores fuera del rango biológicamente posible (n = 17); no tenían registro de estatura (n = 19); no especificaban el sexo (n = 201); y a quienes el tiempo de seguimiento no correspondía con el periodo de tiempo en que fueron levantados los datos (n = 104). Se obtuvo el tiempo de seguimiento a partir de la diferencia de edad entre el segundo y el primer ciclo. El seguimiento más corto fue de dos años para quienes se les encuestó durante el 2006 posterior al día y mes de su nacimiento, con el seguimiento durante el 2009 antes del día y mes en que nacieron; mientras que el máximo fue de ocho años para quienes se levantó la información durante el 2005 antes del día y mes en que nacieron, con el seguimiento durante el 2012 posterior al día y mes de su nacimiento. Se conformó una muestra analítica de 7,130 individuos.

Durante el seguimiento se logró contactar al 81.1% de los participantes incluidos en la ronda inicial. Sin embargo, la medición de presión arterial solo se registró para el 62.7% (n = 4,472). Las mujeres que estaban embarazadas o en periodo de lactancia durante el segundo momento (n = 202) no fueron excluidas, pero solo se incluyó su información basal para el análisis, considerándolas como pérdidas durante el seguimiento. Al comparar los que tenían información completa del seguimiento con quienes solo tenían información de la primera ronda, se identificó que quienes fueron seguidos en general tuvieron menor estatura y mayor presión arterial (Tabla complementaria 1). Por el tipo de análisis, no se excluyeron las pérdidas en el seguimiento, ni a quien tuviera información faltante, ya que se contaba con los resultados de su primera medición.

La presión arterial se midió de la forma establecida en las guías para detección de hipertensión arterial, estando los participantes sentados en un ambiente tranquilo después de al menos cinco minutos de reposo, con el brazo a la altura del corazón, registrando dos mediciones en cada participante^2^. Se obtuvo por medio de un baumanómetro digital marca OMRON modelo HEM^19^, registrándola en mmHg. Para el análisis se utilizó el promedio de las dos mediciones. Se consideraron valores implausibles aquellos con mediciones ≤ 60 o ≥ 300 mmHg para sistólica y ≤ 30 o ≥ 150 mmHg para diastólica. El cambio de la presión arterial se calculó sustrayendo los valores del primer momento de los del segundo momento.

Las medidas antropométricas fueron obtenidas por personal especializado y capacitado para esta actividad, siguiendo procedimientos estandarizados^20^. La medición de la estatura se realizó utilizando un estadímetro de pared marca SECA con precisión de 1.0 mm, estando el individuo de pie, descalzo, recargando talones, pantorrillas, glúteos, espalda y cabeza en la pared. Se reportó en centímetros y se estimaron quintiles para cada sexo.

Fueron consideradas como covariables a la edad de los participantes, la posición socioeconómica, así como hábitos relacionados con la salud y un indicador de adiposidad, la región geográfica y el tamaño de la localidad donde residían. La edad se tomó en años cumplidos registrados en el cuestionario y se agrupó en cinco categorías: 20.0–24.9 años, 25.0–29.9 años, 30.0–34.9 años, 35.0–39.9 años y 40.0 años o más.

Para medir la posición socioeconómica de los participantes se consideró el ingreso familiar y la escolaridad de los padres. El ingreso familiar *per cápita* se obtuvo de la suma de los ingresos de los integrantes de la familia (negocios, rentas, laborales, rurales y transferencias de algún otro familiar o persona). El total se dividió entre el número de miembros del hogar y se reportó en pesos mexicanos. Por otra parte, cuando se disponía de los datos, se analizó la escolaridad del padre; y cuando no se contó con ésta, se consideró la escolaridad de la madre.

Para hábitos (actividad física, consumo de alcohol, tabaquismo y uso de medicamentos antihipertensivos) se generaron variables dicotómicas, distribuyendo la muestra en positivo o negativo de acuerdo con las respuestas. Para actividad física se consideró positivo a quien reportó que realiza actividad física de forma rutinaria. Además, se clasificó positivo a consumo de alcohol quien mencionó que acostumbra ingerir alguna bebida con contenido de alcohol en cualquiera de las situaciones mencionadas en el cuestionario. En tabaquismo, por su parte, se consideró positivo a quien refirió fumar o haber fumado en el pasado. Quien mencionó ser hipertenso y estar con tratamiento se clasificó positivo para uso de antihipertensivos.

La medición de la circunferencia de cintura se realizó con una cinta métrica de fibra de vidrio con 200 cm de longitud y exactitud de un decímetro, estando el individuo de pie, derecho, con los pies juntos, abdomen relajado y brazos cruzados colocados al frente a la altura del pecho. Como referencia anatómica se utilizó la cresta iliaca y la última costilla, la medición se reportó en centímetros^20^. El índice cintura-estatura (ICE) se calculó dividiendo la circunferencia de la cintura sobre la estatura. Este fue considerado como indicador de adiposidad, debido a que ha demostrado ser buen predictor en la evaluación de riesgo cardiovascular. Se consideró como posible mediador de la relación entre estatura y presión arterial ya que las personas con menor estatura tienden a tener mayor adiposidad y, a su vez, la adiposidad es uno de los predictores más fuertes de la presión arterial^21^.

Las regiones se dividieron de acuerdo al plan nacional de desarrollo 2001–2006^22^, quedando cuatro regiones: centro, centro-occidente, sur-sureste y norte (incluyendo noreste y noroeste). De acuerdo con el tamaño de la localidad se formaron cuatro categorías: > 100.000, 15.001–100.000, 2.500–15.000 y < 2.500 habitantes.

La depuración de las bases de datos y el análisis estadístico se realizaron utilizando el programa Stata versión 14. Se consideró el factor de expansión a nivel individual para obtener frecuencias relativas ponderadas de las variables categóricas para cada momento en cada sexo. Se utilizó la prueba ANOVA para buscar diferencias de acuerdo con la estatura en la presión arterial basal y los cambios a lo largo del tiempo.

Para buscar diferencias en la presión arterial (basal y a lo largo del tiempo) de acuerdo con la estatura, se utilizaron modelos multinivel^23^ considerando a la identificación de los participantes como intercepto aleatorio. Con los modelos multinivel se reconoce que existe correlación intra-sujeto. La interacción del ciclo con la estatura permite reconocer las diferencias transversales y longitudinales. El coeficiente de correlación intraclase para la presión sistólica y diastólica en los hombres fue de 0.20 y 0.16; mientras que en las mujeres fue de 0.25 y 0.18, respectivamente. Como variable de tiempo se incluyó el ciclo, que identifica a qué ronda pertenece cada medición. Se agregó el término cuadrático de la estatura y su interacción con el ciclo en cada uno de los modelos para evaluar el efecto cuadrático de la estatura. Se utilizaron modelos crudos y ajustados por datos sociodemográficos, por ICE y por hábitos relacionados con la salud (actividad física, consumo de alcohol, tabaquismo y uso de antihipertensivos). La variable de escolaridad de los padres tuvo una elevada proporción de datos faltantes (mujeres, 15.8%; hombres, 18.2%), por lo cual se estimaron modelos con y sin ella. Para mejorar la comprensión de los resultados, se estimaron y graficaron medias marginales a partir de los modelos ajustados^a^. El análisis se realizó estratificando por sexo.

## RESULTADOS

El 56.1% de los participantes eran mujeres. La edad promedio fue de 29.5 años en un primer momento, aumentando a 35.0 años durante el seguimiento. El tiempo de seguimiento promedio fue de 4.3 años.

En ambos sexos, la mitad de los participantes tenían entre 30 y 39 años durante el 2005 (Tabla 1). Para el segundo momento, la mayoría tenía más de 35 años. La mayor parte de los participantes residían en ciudades y se ubicaron principalmente en la región centro. En los dos ciclos una proporción mayor de la población no realizaba actividad física ni fumaba. La proporción de hombres que realizaba actividad física, fumaba o consumía alcohol fue mayor que la de las mujeres. Para el seguimiento, en los hombres la proporción de fumadores se incrementó, mientras que el consumo de alcohol disminuyó. En las mujeres tanto la proporción de fumadores como el consumo de alcohol aumentaron.

**Tabla 1:** Características de la población de México entre 20 y 45 años^a^ en los años 2005 y 2009.

		2005		2009	
		Hombres (n = 3.133)	Mujeres (n = 3.997)	Hombres (n = 1.734)	Mujeres (n = 2.536)
		%	%	%	%
Edad					
	20 a 24 años	26,9	23,4	3,8	2,4
	25 a 29 años	19,5	19,9	22,2	19,0
	30 a 34 años	26,3	26,7	20,1	18,6
	35 a 39 años	23,0	24,3	25,3	30,6
	40 o más años	4,3	5,8	28,5	29,3
Estrato					
	> 100.000	49,3	47,4	48,5	47,7
	15.001–100.000	12,4	12,6	14,8	14,0
	2.500–15.000	14,7	14,1	14,2	13,5
	< 2.500	23,6	25,9	22,5	24,8
Región geográfica					
	Centro	32,1	33,4	30,6	30,5
	Sur-sureste	21,7	22,4	23,0	23,6
	Centro-Occidente	23,7	21,9	25,5	23,2
	Norte	22,5	22,3	20,9	22,7
Actividad física					
	Positivo	20,0	11,5	22,9	17,2
Tabaquismo					
	Positivo	23,2	8,0	26,9	9,1
Consumo de alcohol					
	Positivo	61,4	18,8	58,5	24,7
		x¯ (SD)	x¯ (SD)	x¯ (SD)	x¯ (SD)
Ingreso familiar*per cápita*		14.268 (23.103)	13.546 (50.358)	15.105 (17.034)	13.332 (20.340)
P. sistólica		116,1 (11,7)	111,2 (11,9)	123,7 (12,9)	112,9 (15,3)
P. diastólica		76,2 (9,7)	72,9 (9,7)	80,1 (9,4)	76,9 (10,9)
Estatura		167,2 (7,6)	154,7 (6,9)	166,6 (7,0)	153,9 (6,8)

n: participantes en la muestra; %: estimación ponderada; x¯: media ponderada; SD: desviación estándar ponderada; P: presión.

a Con el límite de 45 años se cubre la edad de los participantes al momento del seguimiento.

El ingreso familiar *per cápita* fue mayor en los hombres que en las mujeres en los dos ciclos pero, mientras en hombres hubo un incremento en el ingreso promedio, en las mujeres disminuyó ligeramente. La presión sistólica y diastólica promedio en ambos años fue mayor en hombres que en mujeres. De igual forma, el aumento en la presión sistólica entre los hombres fue mayor que en las mujeres, mientras que el aumento de la presión diastólica fue ligeramente mayor en las mujeres. En los hombres el promedio de estatura fue mayor que en las mujeres por 12.5 cm. En el 2009 el promedio de estatura en las mujeres disminuyó 0.8 cm, mientras que en los hombres se redujo a 0.6 cm.

En ambos sexos, la presión arterial basal fue menor en el quintil más bajo de estatura, mientras que la más alta fue en el quintil superior (Tabla 2). Sin embargo, las diferencias fueron significativas solo en los hombres, donde la presión arterial tendió a ser menor conforme el quintil de estatura fue más bajo.

**Tabla 2:** Valores iniciales y cambios entre el 2005 y 2009 en la presión arterial sistólica y diastólica de acuerdo con la estatura de los participantes de la ENNViH de 20 a 45 años^a^.

		Hombres				Mujeres			
		Sistólica		Diastólica		Sistólica		Diastólica	
		Basal	Δ	Basal	Δ	Basal	Δ	Basal	Δ
		M	M	M	M	M	M	M	M
Estatura									
	QV	**118,4** b	8,4	**77,5** b	3,4	112,2	1,0	73,5	3,4
	QIV	**118,7**	6,2	**76,8**	4,1	112,3	1,2	72,7	4,6
	QIII	**116,2**	7,6	**76,4**	3,4	111,1	2,1	72,9	4,0
	QII	**116,8**	8,6	**75,8**	4,9	111,5	2,2	72,3	4,8
	QI	**115,5**	7,3	**75,2**	4,0	110,2	2,3	71,9	4,5

M, media; Basal, valor en 2005; Δ, cambio entre 2005 y 2009; valores en **negritas** implican que existen diferencias significativas en presión entre al menos dos quintiles de estatura.

a Con el límite de 45 años se cubre la edad de los participantes al momento del seguimiento.

b p < 0.001.

**Tabla 3:** Modelos multinivel considerando la presión arterial sistólica y diastólica como variables dependientes y la estatura como variable independiente en hombres de 20 a 45 años^a^ participantes de la ENNViH (2005, 2009).

		Mod. 0		Mod. 1		Mod. 2		Mod. 3	
		β	E.E	β	E.E	β	E.E	β	E.E
Sistólica									
	Estatura	**0,143**	**0,030**	**0,111**	**0,031**	**0,151**	**0,030**	**0,159**	**0,032**
	Estatura*estatura	-0,003	0,003	-0,002	0,003	-0,002	0,003	-0,001	0,003
	Ciclo	**7,487**	**0,425**	**6,460**	**0,452**	**5,940**	**0,449**	**5,978**	**0,467**
	Ciclo*estatura	0,073	0,048	0,064	0,048	0,065	0,047	0,028	0,049
	Ciclo*estatura*estatura	**0,009**	**0,004**	**0,009**	**0,004**	**0,009**	**0,004**	**0,010**	**0,004**
Diastólica									
	Estatura	**0,099**	**0,023**	**0,090**	**0,024**	**0,129**	**0,024**	**0,122**	**0,025**
	Estatura*estatura	-0,002	0,002	-0,002	0,002	-0,002	0,002	-0,002	0,002
	Ciclo	**3,785**	**0,342**	**2,914**	**0,361**	**2,375**	**0,356**	**2,320**	**0,370**
	Ciclo*estatura	-0,016	0,039	-0,018	0,039	-0,018	0,038	-0,023	0,040
	Ciclo*estatura*estatura	0,006	0,004	0,006	0,004	**0,006**	**0,004**	**0,008**	**0,004**

β, Coeficiente de regresión; E.E, error estándar. valores en **negritas** implican que existen diferencias significativas. Para los efectos principales se utilizó un nivel de significancia del 0.05 (p < 0.05) y para las interacciones el nivel de significancia fue de 0.10 (p < 0.10). Mod. 0, modelo sin ajustar por otras variables. Mod. 1, modelo ajustado por edad, estrato, región e ingreso familiar *per cápita*. Mod. 2, modelo ajustado por las variables del modelo 1 e índice cintura-estatura. Mod. 3, modelo ajustado por las variables del modelo 2 más actividad física, alcoholismo, tabaquismo. y uso de antihipertensivos.

a Con el límite de 45 años se cubre la edad de los participantes al momento del seguimiento.

En cada uno de los modelos multinivel (Tablas 3 y 4), en ambos sexos, la presión sistólica y diastólica fueron más altas en el seguimiento en comparación con la medición basal, excepto en la presión sistólica en las mujeres, donde esta diferencia fue significativa solamente en el modelo crudo (modelo 0). En los hombres (Tabla 3 y figura suplementaria), ambas presiones, al inicio del seguimiento, tendieron a ser menores conforme la estatura fuese más baja. Esta tendencia se observó tanto en los modelos crudos como en los ajustados por diferentes covariables (modelos 0 al 3). Al ajustar por ICE esta relación se hizo más fuerte (modelo 2), tanto en sistólica como en diastólica. Evaluando el efecto cuadrático se identificó que los incrementos de la presión sistólica a lo largo del tiempo tendieron a ser mayores conforme la estatura era más alta, en cada uno de los modelos (modelos 0 al 3). El efecto en la presión diastólica fue similar pero solamente al ajustar por ICE y todas las variables (modelos 2 y 3).

**Tabla 4:** Modelos multinivel considerando la presión arterial sistólica y diastólica como variables dependientes y la estatura como variable independiente en mujeres de 20 a 45 años^a^ participantes de la ENNViH (2005, 2009).

		Mod. 0		Mod. 1		Mod. 2		Mod. 3	
		β	E.E	β	E.E	β	E.E	β	E.E
Sistólica									
	Estatura	**0,069**	**0,027**	**0,085**	**0,029**	**0,163**	**0,029**	**0,165**	**0,030**
	Ciclo	**2,394**	**0,328**	0,428	0,348	0,156	0,345	0,298	0,355
	Ciclo*estatura	-0,062	0,049	-0,062	0,049	-0,045	0,048	-0,039	0,049
Diastólica									
	Estatura	0,034	0,021	**0,045**	**0,022**	**0,116**	**0,023**	**0,113**	**0,023**
	Ciclo	**4,677**	**0,240**	**3,510**	**0,259**	**3,219**	**0,257**	**3,292**	**0,263**
	Ciclo*estatura	-0,048	0,035	-0,045	0,035	-0,028	0,034	-0,029	0,035

β, Coeficiente de regresión; E.E, error estándar. valores en **negritas** implican que existen diferencias significativas. Para los efectos principales se utilizó un nivel de significancia del 0.05 (p < 0.05) y para las interacciones el nivel de significancia fue de 0.10 (p < 0.10). Mod. 0, modelo sin ajustar por otras variables. Mod. 1, modelo ajustado por edad, estrato, región e ingreso familiar *per cápita*. Mod. 2, modelo ajustado por las variables del modelo 1 e índice cintura-estatura. Mod. 3, modelo ajustado por las variables del modelo 2 más actividad física, alcoholismo, tabaquismo y uso de antihipertensivos.

a Con el límite de 45 años se cubre la edad de los participantes al momento del seguimiento.

En las mujeres (Tabla 4 y figura suplementaria), las presiones basales también fueron menores conforme la estatura fuese más baja, especialmente después de ajustar por ICE y por los hábitos relacionados con la salud (modelos 2 y 3). No se identificaron diferencias en los cambios a lo largo del tiempo, ni al utilizar términos cuadráticos (Tabla suplementaria 2).

Los resultados de los modelos al ajustar por la escolaridad de los padres (Tablas suplementarias 3 y 4) no difirieron de los obtenidos sin incluirla (Tablas 3 y 4). Por ello, sólo se reportaron en el manuscrito los últimos.

## DISCUSIÓN

Considerando la evidencia epidemiológica de otras poblaciones^3–5,8–11^ y los cambios anatómicos y fisiológicos que se han descrito^13,14^, se esperaría una relación inversa entre la estatura y la presión arterial inicial. Sin embargo, en adultos mexicanos la presión arterial basal tendió a ser mayor conforme la estatura fue más alta en ambos sexos, con diferencias más amplias entre los hombres que entre las mujeres. En los cambios de la presión arterial, solamente se encontraron diferencias en los hombres al evaluar los efectos cuadráticos, contrario a lo esperado, conforme la estatura fue mayor, el incremento de la presión arterial fue más alto.

Las diferencias en la composición genética de la población podrían ayudar a explicar porque el comportamiento de la presión arterial no fue como se esperaba. El mestizaje de nuestro país resultó de la combinación de tres ancestrías: amerindia, europea y africana. La ascendencia europea se ha asociado con mayor estatura^17^, así como con mayor riesgo de presentar enfermedades cardiovasculares^24^, en comparación con la ascendencia amerindia. Por el contrario, el tener ancestría amerindia se relaciona con mayor probabilidad de presentar estatura baja^25^. Es decir, es posible que la población euro descendiente de México es la que tiene mayor estatura y presión arterial respecto al resto de la población y eso es lo que explica la relación positiva entre esas dos variables observadas en nuestro análisis. Desafortunadamente en la ENNVIH no se midieron marcadores de la ancestría por lo cual no es posible evaluar esta posibilidad. Por otro lado, la relación de la presión arterial y la estatura podría no ser lineal, por lo cual, en poblaciones de menor estatura en comparación a países europeos el comportamiento sea contrario al que se ha reportado en poblaciones de mayor estatura.

Además de la genética, la calidad de la dieta podría ser otro factor que ayude a explicar porque los participantes de mayor estatura tuvieron mayor presión arterial. En México, la disponibilidad y el consumo de alimentos ultraprocesados tienen diferencias de acuerdo a la región geográfica y nivel socioeconómico (similares a lo que ocurre con la estatura), habiendo mayor consumo en el norte y por las personas con mejor nivel socioeconómico^26^. Los alimentos ultraprocesados tienen más alto contenido de sodio en comparación con los de origen natural^26,27^, lo cual explica porque su consumo se asocia con elevación de la presión arterial^27^. Por lo tanto, es posible que las personas con mayor estatura tiendan a tener nivel socioeconómico más alto^28^, por lo cual pueden tener mayor consumo de alimentos ultraprocesados, favoreciendo que la presión arterial sea más alta conforme la estatura sea mayor. Esta posibilidad no fue explorada ya que en la ENNViH no se registró información relacionada con dieta.

La participación del ICE como mediador en la relación entre la estatura y la presión arterial se confirmó. El ICE se relacionó inversamente con la estatura, quienes tenían menor estatura tuvieron mayor ICE, tal como se reportó en un estudio realizado en el sur de México^25^. En los modelos donde no se incluyó, las diferencias de acuerdo con la estatura fueron menores pero, al agregarlo a los modelos, la relación de la estatura con la presión sistólica y diastólica basal se hizo más fuerte^21^.

Dentro de las limitaciones del estudio es importante señalar que la ENNViH no fue diseñada para probar nuestra hipótesis, por lo que fue necesario adaptar la información disponible. Además, a excepción de las medidas antropométricas, la información se obtuvo a través de cuestionarios. El tiempo de seguimiento promedio de este estudio fue de cuatro años, mientras que varios de los estudios realizados cuentan con un tiempo de seguimiento superior a 15 años^5,6,11^. Finalmente, cabe la posibilidad de que los cambios de la presión arterial a lo largo del tiempo sean significativos en otra etapa de la vida. Es decir, probablemente no se identificaron diferencias en los cambios de la presión arterial debido a que el periodo de seguimiento fue en un momento en el que la presión arterial se eleva uniformemente, independientemente de la estatura. Las diferencias podrían identificarse en etapas más tempranas^6^, o bien, en edades posteriores a las incluidas en la investigación^4^.

A pesar de las limitaciones antes mencionadas, este estudio permitió tener un panorama general de la asociación entre la estatura con la presión arterial en la población mexicana. La presión arterial se midió siguiendo las recomendaciones de las guías de detección de hipertensión arterial^2^. Aunque podría ser útil contar con más de dos mediciones^29^, el incremento en el número de mediciones ha significado un mínimo beneficio en la detección de riesgo cardiovascular^30^. Además, se analizaron datos de un estudio con representatividad nacional y diseño longitudinal. El empleo de modelos multinivel permitió distinguir entre diferencias transversales de cambios a lo largo del tiempo. Por lo tanto, las relaciones encontradas son causalmente más sólidas que las que podrían haberse encontrado en un estudio transversal.

En resumen, en la población mexicana, el efecto de la estatura sobre la presión arterial fue contrario a lo que se esperaba, ya que se relacionó de forma positiva. En el contexto de la transición epidemiológica, se ha propuesto que el predominio de la desnutrición crónica en las poblaciones latinoamericanas podría ser un factor que incremente la carga asociada a las enfermedades crónicas. Nuestro análisis muestra que esto podría no ser completamente cierto. En contraste con lo anterior, en los adultos mexicanos la presión arterial se relacionó en la dirección esperada con factores de riesgo tradicionales como la adiposidad. Por ello, para enfrentar la problemática de la hipertensión arterial y sus consecuencias en el país, es necesario continuar investigando otros factores ambientales, genéticos y dietéticos; así como atender los factores modificables ya conocidos. Convendría realizar estudios con un diseño similar, pero en diferentes etapas de la vida, que permitan evaluar el comportamiento de la presión arterial e identificar los momentos en que se presenta un mayor cambio.

## Footnotes

a La Figura suplementaria puede ser solicitada a los autores.
